# The relationship between perceived social support and health-promoting behaviors among middle-aged and young patients after PCI: the mediating role of fear of disease progression

**DOI:** 10.3389/fpubh.2026.1839180

**Published:** 2026-10-21

**Authors:** Yuxin Li, Xiaoli Zhong, Tianxia Zhao, Ping Dai, Junrong Yang, Yuting Fan, Jijun Wu, Lin He

**Affiliations:** 1 Department of Nursing, Deyang People’s Hospital, Deyang, Sichuan, China; 2 Department of Cardiology, Deyang People’s Hospital, Deyang, Sichuan, China

**Keywords:** fear of disease progression, health-promoting behaviors, mediating effect, middle-aged and young adults, perceived social support, percutaneous coronary intervention

## Abstract

**Objective:**

This study aims to investigate the mediating role of fear of disease progression in the relationship between perceived social support and health-promoting behaviors among middle-aged and young patients after PCI, providing a theoretical basis for developing targeted interventions to improve health promotion behaviors in this population.

**Methods:**

This cross-sectional study employed convenience sampling. Between April and October 2024, 265 middle-aged and young patients who underwent PCI were surveyed in the cardiovascular department of a Grade A tertiary hospital in Sichuan Province, China. Data collection utilized questionnaires on general information, the Health-Promoting Lifestyle Scale, the Perceived Social Support Scale, and the Fear of Disease Progression Scale. Data analysis was performed using SPSS 26.0 software for descriptive analysis, univariate analysis, correlation analysis, and multivariate stratified regression analysis. Mediating effect analysis was conducted using Model 4 in the Process v4.1 plugin.

**Results:**

The mean health-promoting behaviors score among middle-aged and young patients after PCI was (88.09 ± 9.98) points. Correlation analysis revealed that perceived social support was negatively correlated with fear of disease progression (*r* = −0.478, *p* < 0.01) and positively correlated with health-promoting behaviors (*r* = 0.715, *p* < 0.01). Fear of disease progression was negatively correlated with health-promoting behaviors (*r* = −0.610, *p* < 0.01). Mediation analysis revealed that fear of disease progression mediated the relationship between perceived social support and health-promoting behaviors, with an effect size of 0.068. This mediation accounted for 13.9% of the total effect.

**Conclusion:**

There is still room for further improvement in health-promoting behaviors among middle-aged and young patients following PCI. There is a significant correlation among perceived social support, fear of disease progression, and health-promoting behaviors, with fear of disease progression partially mediating the relationship between perceived social support and health-promoting behaviors. This study elucidates the interrelationships among these three factors and their underlying mechanisms, suggesting that clinicians may develop personalized rehabilitation management plans by integrating patients’ social support status with their psychological characteristics regarding disease-related fears. This approach offers new insights for optimizing health behavior management in middle-aged and young patients following PCI.

## 1 Introduction

Coronary heart disease (CHD) is the leading cause of cardiovascular disease mortality worldwide, posing a major public health challenge that seriously threatens public health and exacerbates the social burden of healthcare. Survey data indicate that as of 2019, approximately 197 million people globally suffered from CHD, with the prevalence projected to increase by 18.0% by 2030 (1, 2). Data from the 2024 China Cardiovascular Health and Disease Annual Report also indicates that the prevalence of CHD among adults aged 18 and above in China reached 758 cases per 100,000 population. Notably, the prevalence rate among males (940 cases per 100,000) was significantly higher than that among females (570 cases per 100,000) (3). Alarming trends indicate that in recent years, influenced by high-salt, high-fat diets, smoking, lack of exercise, and the high prevalence of metabolic diseases, CHD onset in China has shown a marked trend toward younger age groups. The number of middle-aged and young CHD patients continues to rise, making them a key focus group for cardiovascular disease prevention and control (4). Percutaneous coronary intervention (PCI) has become the preferred treatment for middle-aged and young CHD patients due to its advantages of minimal trauma, rapid revascularization, and clear symptom relief, significantly reducing the incidence of acute cardiovascular events and short-term mortality (5). However, PCI only resolves localized vascular stenosis and cannot reverse the pathophysiological progression of coronary heart disease. Postoperative risks persist, including in-stent restenosis, thrombosis, recurrent angina, and even cardiac death (3). Studies indicate that the incidence of adverse cardiovascular events after PCI remains high at 10–20%, severely impacting patients’ long-term prognosis, quality of life, and restoration of social functioning (6, 7). As the core bearers of social productivity and family responsibilities, the health status of middle-aged and young adults after PCI not only concerns individual well-being but also profoundly impacts family functioning and societal productivity. Therefore, exploring key pathways to enhance post-PCI health management for this demographic holds significant practical importance.

Health-Promoting Behavior (HPB) play a critical role in secondary prevention for coronary heart disease. Encompassing multidimensional aspects such as balanced nutrition, regular exercise, standardized medication use, periodic follow-up examinations, emotional management, and risk factor control, these behaviors are decisive factors in reducing postoperative complications, improving long-term prognosis, and enhancing quality of life (8). It is a key factor in improving the long-term prognosis of patients following PCI procedures. Research indicates that robust HPB effectively reduce the incidence of adverse cardiovascular events—such as in-stent restenosis, arrhythmia, and heart failure—in PCI patients, decrease readmission rates, and significantly enhance patients’ quality of life and long-term survival (9). However, existing research indicates that young and middle-aged PCI patients generally exhibit low levels of HPB and poor adherence to guidelines due to factors such as high work stress, health cognition biases, and inadequate self-management capabilities (10). This makes it difficult for them to sustain standardized postoperative health management over the long term. Furthermore, studies indicate that these patients commonly exhibit insufficient health awareness, poor medication adherence, and recurrence of unhealthy lifestyle habits (11). These issues not only compromise postoperative recovery outcomes and increase the risk of recurrent cardiovascular events but also adversely affect patients’ psychological well-being, triggering negative emotions such as anxiety and depression, thereby exacerbating health deterioration. Therefore, in-depth exploration of the influencing factors and mechanisms underlying HPB among young and middle-aged patients after PCI is crucial for improving their HPB and long-term rehabilitation outcomes, as well as alleviating the medical burden on families and society.

Perceived Social Support (PSS), as the subjective dimension of social support, refers to an individual’s cognition, experience, and evaluation of external support from family, friends, healthcare providers, and others (12). Its core value lies in alleviating psychological stress and enhancing an individual’s resources and confidence in coping with illness. Existing research indicates that PSS plays a significant positive role in regulating health behaviors among patients with chronic diseases (13). Higher levels of PSS can promote proactive HPB by enhancing psychological resilience and reducing anxiety and depressive symptoms. For middle-aged and young patients post-PCI, their complex social roles and diverse psychological needs suggest that emotional support from family, companionship from friends, and professional support from healthcare providers could theoretically offer psychological reinforcement and behavioral guidance for postoperative recovery, helping them overcome challenges during rehabilitation (14). However, previous studies indicate that young and middle-aged patients after PCI often experience low PSS due to factors such as busy work schedules hindering effective communication with loved ones, avoidance of expressing emotional needs out of fear of becoming a family burden, and insufficiently targeted follow-up support from medical staff (15). Furthermore, research confirms that patients with higher levels of PSS experience greater psychological stress relief, stronger confidence in recovery, more proactive engagement with health guidance, greater willingness to avoid unhealthy lifestyles, and increased likelihood of adopting beneficial HPB (16).

Fear of Disease Progression (FoP) is a common negative psychological state among patients with chronic diseases. It refers to excessive worry and fear regarding potential consequences such as disease progression, functional loss, death, and associated impairments in social functioning (17). At its core, FoP reflects an individual’s subjective cognitive appraisal of disease-related threats and future uncertainty. Patients who have undergone PCI may experience FoP because of the chronic nature of coronary artery disease, the ongoing need for medical management following stent implantation, and concerns about disease recurrence. This may be particularly relevant to young and middle-aged patients, who are often at an important stage of fulfilling family responsibilities, developing their careers, and undertaking social roles; concerns about disease recurrence and the prospect of long-term living with the disease may be associated with greater anxiety about future health and social functioning (18). Previous studies have identified social support as an important psychosocial factor associated with FoP in patients with chronic conditions. Higher levels of social support are associated with greater access to emotional comfort, disease-related information, and practical assistance, as well as greater coping resources and perceived control. These factors may, in turn, be associated with lower disease-related uncertainty and perceived threat and with lower levels of FoP (19). Conversely, FoP may also be associated with patients’ health behaviors. When patients have greater concerns about disease recurrence or progression, they may exhibit greater avoidance of activities perceived as potentially unsafe, such as exercise and dietary changes, or may report lower engagement in disease management and health-promoting activities in the context of helplessness or low self-efficacy (20, 21).

Social Cognitive Theory (SCT), proposed by Bandura in 1986, posits that individual behavior results from the reciprocal interaction among personal factors, environmental factors, and behavior (22). Within this framework, environmental factors may be related to health behaviors both directly and indirectly through their associations with individuals’ cognitive appraisal and processing of relevant information and disease-related threats (12). In the present study, PSS can be conceptualized as an external social environmental resource perceived by the individual. Emotional, informational, and practical support from family members, friends, and significant others may be associated with greater coping resources and perceived control over illness and with lower levels of disease-related uncertainty (16). FoP primarily reflects an individual’s subjective appraisal of threats related to disease recurrence, progression, and potential physical, psychological, and social consequences, and involves both cognitive and emotional responses (17). Among young and middle-aged patients after PCI, higher levels of perceived social support may be associated with lower levels of FoP, potentially because social support provides access to disease-related information, emotional comfort, and practical assistance and is related to greater perceived control and coping resources. Conversely, lower levels of PSS may be associated with greater uncertainty and perceived threat regarding the future course of the disease and, consequently, with higher levels of fear of disease progression. At the same time, fear of disease progression, as a cognitive-emotional response to perceived disease-related threats, may be associated with patients’ behavioral choices. Higher levels of FoP may be associated with greater avoidance or passive coping tendencies and lower engagement in exercise, healthy dietary practices, health responsibility, and other HPB, whereas lower levels of fear of disease progression may be associated with greater engagement in positive disease management and HPB (23). Therefore, from the perspective of SCT, FoP may represent a relevant cognitive-emotional factor linking perceived environmental resources with HPB.

In summary, based on SCT, this study conceptualizes perceived social support as an external environmental factor, fear of disease progression as a cognitive-emotional response to perceived disease-related threats, and HPB as behavioral outcomes. Accordingly, we proposed the potential pathway “PSS → FoP → HPB” and examined the potential mediating role of FoP in the association between PSS and HPB. This framework provides a theoretical basis for exploring the relationships among PSS, FoP, and HPB in young and middle-aged patients after PCI, while recognizing that the cross-sectional design does not establish causal relationships or underlying mechanisms (Figure 1).

**Figure 1:**
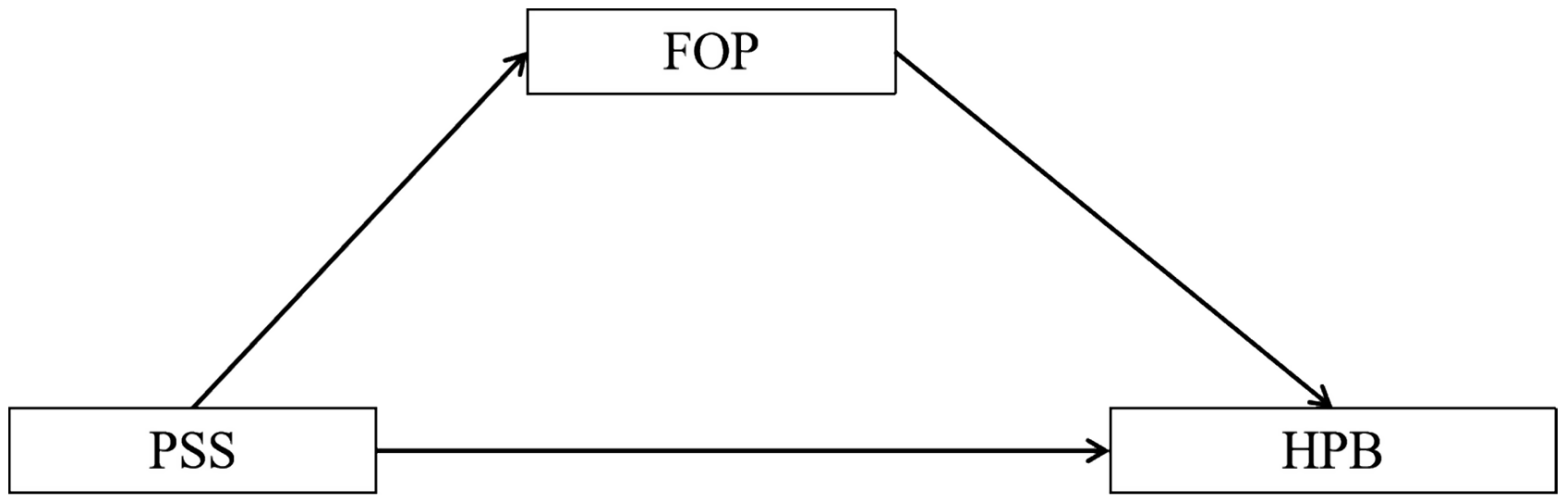
Research hypothesis mediation model.

## 2 Methods

### 2.1 Study design

This study is a cross-sectional study.

### 2.2 Participants

Using convenience sampling, middle-aged and young patients undergoing PCI procedures in the Cardiovascular Department of a Grade A tertiary hospital in Sichuan Province, China, were selected as study subjects from April to October 2024. This study primarily reflects the characteristics of young and middle-aged patients following PCI in a Grade A tertiary hospital in Sichuan Province, China. It is a single-center observational study. All participants were recruited from this medical institution. Inclusion criteria: ① Patients meeting diagnostic and treatment criteria for coronary heart disease (24), fulfilling indications for PCI, and in stable physical condition post-PCI; ② Patients aged 18–59 years; ③ Patients with clear consciousness, normal cognitive function, and the ability to communicate verbally or in writing; ④ Patients who provided informed consent and voluntarily participated in the study. Exclusion criteria: ① Patients with severe concomitant systemic diseases, such as liver, brain, or kidney damage, tumor recurrence or metastasis, or severe infectious diseases; ② Patients unable to complete the study survey for any reason (e.g., severe visual/auditory impairment, aphasia, altered consciousness). Sample size was calculated using G*Power software (version 3.1) (25). Based on Cohen’s standards (26), power was set at 0.95, alpha at 0.05, and effect size f^2^ at 0.15 (medium effect size). This study involved 16 independent variables. Based on these parameters, G*Power calculated a required sample size of 204. Accounting for a 10% non-response rate, the sample size was adjusted to 227. Ultimately, 265 participants were enrolled, meeting the requirements outlined above.

### 2.3 Measures

#### 2.3.1 General information questionnaire

A self-designed general information questionnaire was administered, covering gender, age, marital status, number of children, education level, employment status, place of residence, medical payment methods, average monthly family income, number of chronic diseases, disease duration, number of interventional treatments, number of stent implants.

#### 2.3.2 Health-promoting lifestyle scale

This study employed the Health-Promoting Lifestyle Scale developed by Walker et al. (27) and translated into Chinese by Cao et al. (28) to assess individuals’ HPB. This scale comprises six dimensions—nutrition, physical activity, health responsibility, interpersonal relationships, spiritual growth, and stress management—totaling 40 items. It uses a 4-point Likert scale, yielding a total score of 40–160. Responses from “Never” to “Always” are scored as 1 to 4 points, respectively, with higher scores indicating better HPB. The scale demonstrates excellent reliability and validity, with a Cronbach’s α coefficient of 0.930. In this study, the Cronbach’s α coefficient for the scale was 0.915, indicating good internal consistency among patients who underwent PCI in this study.

#### 2.3.3 Perceived social support scale

This study employed the Perceived Social Support Scale developed by Blumenthal et al. (29) and adapted into Chinese by Jiang et al. (30) to assess individuals’ levels of perceived social support. This scale comprises three dimensions—family support, friend support, and other support—totaling 12 items. It employs a 7-point Likert scale ranging from “strongly disagree” to “strongly agree,” scored from 1 to 7. The total score ranges from 12 to 84 points, with higher scores indicating greater perceived levels of social support. This scale demonstrates excellent reliability and validity and is widely used to assess PSS among patients with chronic diseases. Its Cronbach’s α coefficient is 0.840. In this study, the Cronbach’s α coefficient for this scale was 0.886, indicating good internal consistency among patients who underwent PCI in this study.

#### 2.3.4 Fear of disease progression scale

This study employed the Fear of Disease Progression Scale developed by Mehnert et al. (31) and adapted into Chinese by Wu et al. (32) to assess individuals’ levels of fear of disease progression. This scale comprises 12 items across two dimensions: physical health and social/family. It employs a 5-point Likert scale ranging from “never” to “always,” scored 1–5, yielding a total score of 12–60. Higher scores indicate a more severe FoP. This scale demonstrates good reliability and validity and is widely used to assess FoP in patients with chronic diseases. Its Cronbach’s α coefficient is 0.883. In this study, the Cronbach’s α coefficient for this scale was 0.827, indicating good internal consistency among patients who underwent PCI in this study.

### 2.4 Data collection

This study employed paper-based questionnaires as the primary data collection tool. Prior to formal implementation, three investigators underwent standardized specialized training covering questionnaire completion requirements, survey procedures, and quality control essentials to ensure process standardization. Following training, research subjects were screened according to predefined inclusion and exclusion criteria. During the survey, investigators used standardized instructions to explain the study’s purpose, significance, completion process, and relevant precautions to participants. Questionnaires were distributed only after obtaining informed consent and signed written informed consent forms. For participants unable to complete the questionnaire independently, investigators read each item aloud in a neutral manner and assisted with completion. Disease-related information for participants was collected and verified through electronic medical record reviews and face-to-face interviews. Throughout questionnaire completion, investigators avoided providing leading prompts or interference to ensure data authenticity and reliability. Completed questionnaires were collected on-site and immediately checked for completeness and logical consistency. Any missing or erroneous entries were promptly supplemented or corrected. A total of 278 questionnaires were distributed. After individual review, 13 invalid questionnaires—including those with logical inconsistencies or patterned responses—were excluded, yielding 265 valid responses. The effective recovery rate was 95.3%.

### 2.5 Statistical methods

Statistical analyses were performed using SPSS 26.0. First, the Harman single-factor test was used to assess common method bias. All measurement items were included in exploratory factor analysis; if the first principal component extracted without rotation explained less than 40% of the variance, common method bias was considered absent. Quantitative data following a normal distribution were described as mean ± standard deviation, while categorical data were described using frequencies and proportions. Intergroup comparisons were performed using the independent samples t-test or one-way analysis of variance (ANOVA). Pearson correlation analysis was used to examine the relationships among variables. Hierarchical multiple linear regression was employed to analyze the determinants of HPB and the mediating role of FoP. Prior to regression analysis, multicollinearity was assessed using the Variance Inflation Factor (VIF) and Tolerance. Model 4 in the Process v4.1 plugin was used to test the indirect effect of FoP on the relationship between PSS and HPB. Bootstrap sampling was performed with 5,000 repetitions; a 95% confidence interval (CI) excluding 0 indicated a statistically significant effect. The significance level was set at a two-sided α = 0.05.

### 2.6 Ethical considerations

The study followed the Declaration of Helsinki and was approved by the Ethics Committee of Deyang People’s Hospital (2024–04-018-K01). All study subjects gave informed consent and voluntarily participated in this study.

## 3 Results

### 3.1 General characteristics of young and middle-aged patients after PCI

Among the 265 young and middle-aged patients who underwent PCI in this study, 158 (59.6%) were male and 107 (40.4%) were female. The majority were aged 45–59 years (85.3%), and most were married (89.8%). Additional general characteristics are shown in Table 1.

**Table 1:** Relationship between general characteristics and HPB among middle-aged and young patients after PCI.

Items	N(%)	Mean ± SD	*t/F*	*p*
Gender			−0.279	0.780
Male	158(59.6)	87.95 ± 9.55		
Female	107(40.4)	88.30 ± 10.63		
Age (years)			−10.306	<0.001
18 ~ 44	39(14.7)	75.23 ± 9.42		
45 ~ 59	226(85.3)	90.31 ± 8.26		
Marital status			5.717	<0.001
Married	238(89.8)	89.21 ± 9.40		
Single/Divorced/Widowed	27(10.2)	78.26 ± 9.69		
Number of children			0.694	0.501
0	2(0.8)	95.00 ± 8.49		
1	194(73.2)	87.80 ± 10.68		
≥2	69(26.0)	88.71 ± 7.74		
Education level			38.201	<0.001
Elementary and below	109(41.1)	83.56 ± 8.97		
Junior	90(34.0)	88.09 ± 9.92		
High school and above	66(24.9)	95.58 ± 6.68		
Employment Status			1.185	0.237
Unemployed	99(37.4)	89.03 ± 9.64		
Employed	166(62.6)	87.53 ± 10.16		
Place of residence			8.116	<0.001
Towns	148(55.8)	92.05 ± 8.68		
Countryside	117(44.2)	83.08 ± 9.26		
Medical payment methods			36.903	<0.001
Urban medical insurance	125(47.2)	93.03 ± 8.07		
Rural medical insurance	91(34.3)	83.63 ± 8.90		
Self-funded	49(18.5)	83.78 ± 10.51		
Average monthly family income(yuan)			48.116	<0.001
<3,000	22(8.3)	79.45 ± 12.44		
3,000–<5,000	154(58.1)	85.33 ± 8.79		
≥5,000	89(33.6)	95.00 ± 6.88		
Number of chronic diseases			15.562	<0.001
1	113(42.6)	91.66 ± 8.96		
2	107(40.4)	86.31 ± 9.24		
≥3	45(17.0)	83.36 ± 11.12		
Disease duration(years)			6.985	<0.001
<3	175(66.0)	89.66 ± 9.71		
3–<5	69(26.0)	84.59 ± 9.62		
≥5	21(7.9)	86.48 ± 10.64		
Number of interventional treatments			27.431	<0.001
1	185(69.8)	90.73 ± 8.77		
2	69(26.0)	82.74 ± 10.24		
≥3	11(4.2)	77.27 ± 6.86		
Number of stent implants			18.056	<0.001
1	98(37.0)	91.60 ± 9.19		
2	94(35.5)	88.46 ± 8.45		
≥3	73(27.5)	82.90 ± 10.73		

### 3.2 Univariate analysis of HPB among young and middle-aged patients after PCI

Univariate analysis revealed statistically significant differences (*p* < 0.05) in HPB scores among young and middle-aged patients after PCI across various factors: age, marital status, education level, place of residence, medical payment methods, average monthly family income, number of chronic diseases, disease duration, number of interventional treatments, number of stent implants. See Table 1.

### 3.3 Common method bias test

To further enhance the rigor of this study, the Harman single-factor analysis method was employed to conduct an unrotated exploratory factor analysis on all scale items. The results revealed 15 factors with eigenvalues greater than 1 without rotation. The first factor explained 23.74% of the variance, falling below the 40% critical threshold. Therefore, it can be concluded that no significant common method bias exists in this study.

### 3.4 Descriptive and correlation analysis of scales

In this study, middle-aged and young patients who underwent PCI achieved PSS scores of 49.96 ± 4.69, FoP scores of 33.30 ± 4.98, and HPB scores of 88.09 ± 9.98. Correlation analysis revealed that PSS was negatively correlated with FoP (*r* = −0.478, *p* < 0.01), positively correlated with HPB (*r* = 0.715, *p* < 0.01), and that FoP was negatively correlated with HPB (*r* = −0.610, *p* < 0.01). See Table 2.

**Table 2:** Descriptive and correlation analysis of PSS, FoP, and HPB.

Variables	Mean ± SD	1	2	3
1PSS	49.96 ± 4.69	1		
2FoP	33.30 ± 4.98	−0.478*	1	
3HPB	88.09 ± 9.98	0.715*	−0.610*	1

^*^p < 0.01.

### 3.5 Hierarchical multiple linear regression analysis

First, based on the results of the univariate analysis, variables with statistically significant associations with health-promoting behaviors were included as candidate variables; subsequently, a stratified multiple linear regression analysis was conducted to further identify factors independently associated with HPB. In the first step, variables that were statistically significant in the univariate analysis were included as control variables; in the second step, PSS was added to this model; and in the third step, FoP was further included. The results showed that after including the relevant control variables, Model 1 had an R^2^ of 0.607 and an adjusted R^2^ of 0.591; After adding PSS, the R^2^ of Model 2 increased to 0.686, an increase of 0.079 compared to Model 1, representing a 7.9% increase in the proportion of variance explained; moreover, PSS was positively associated with HPB (*β* = 0.406, *p* < 0.001). Upon further inclusion of FoP, the R^2^ of Model 3 increased to 0.711, an increase of 0.025 compared to Model 2, representing a further 2.5% increase in the proportion of variance explained; At this point, the regression coefficient for PSS decreased from 0.406 to 0.366 but remained statistically significant (*p* < 0.001), while FoP was negatively associated with HPB (*β* = −0.199, *p* < 0.001). The R^2^ values for each model ranged from 0.385 to 0.851, and the variance inflation factors ranged from 1.174 to 2.599; no significant multicollinearity was observed. Finally, the statistically significant variables from the stratified multiple linear regression analysis were included as covariates in the subsequent mediation analysis. See Table 3.

**Table 3:** Multivariate stratified regression analysis of HPB among middle-aged and young patients after PCI.

Variables	Model 1			Model 2			Model 3		
	*β*	*t*	*p*	*β*	*t*	*p*	*β*	*t*	*p*
Control variables									
Age	0.393	9.366	<0.001	0.314	8.085	<0.001	0.271	7.043	<0.001
Education level	0.224	4.733	<0.001	0.110	2.458	0.015	0.090	2.090	0.038
Medical payment methods	−0.090	−1.801	0.073	−0.133	−2.956	0.003	−0.121	−2.795	0.006
PSS				0.406	8.014	<0.001	0.366	7.412	<0.001
FoP							−0.199	−4.669	<0.001
F		39.204**			50.349**			51.765**	
R^2^		0.607			0.686			0.711	
Adjusted R^2^		0.591			0.673			0.698	

^**^p < 0.001; β represents standardized regression coefficients.

Note: In Model 1, all variables that were statistically significant in the univariate analysis (including age, marital status, education level, place of residence, medical payment methods, average monthly family income, number of chronic diseases, disease duration, number of interventional treatments, number of stent implants) were included as covariates. The table shows only those variables that reached statistical significance.

### 3.6 Testing the mediating effect of FoP on the relationship between PSS and HPB in young and middle-aged patients after PCI

All variables were standardized. Using PSS as the independent variable, health promotion behaviors as the dependent variable, FoP as the mediating variable, and age, educational level, and medical payment methods as control variables, mediation analysis was conducted using Model 4 in Process v4.1.

The mediation analysis results revealed that PSS positively predicted FoP: a = −0.303, SE = 0.064, *p* < 0.001. PSS positively predicted HPB, c’ = 0.421, SE = 0.044, *p* < 0.001; FoP negatively predicted health promotion behaviors, b = −0.223, SE = 0.041, *p* < 0.001. The Bootstrap method revealed that FoP partially mediated the positive relationship between PSS and HPB, with ab = 0.068, BootSE = 0.021, and a 95% CI of [0.032, 0.114]. The mediation effect accounted for 13.9% of the total effect. See Table 4 and Figure 2.

**Table 4:** Results of the mediating effect analysis.

Effects	Effect size	SE	95% CI
Total effect			
PSS → HPB	0.488	0.044	(0.401, 0.576)
Direct effect			
PSS → HPB	0.421	0.044	(0.334, 0.507)
Indirect effect			
PSS → FoP → HPB	0.068	0.021	(0.032, 0.114)

The mediation analysis controlled for age, educational level, and medical payment methods.

**Figure 2:**
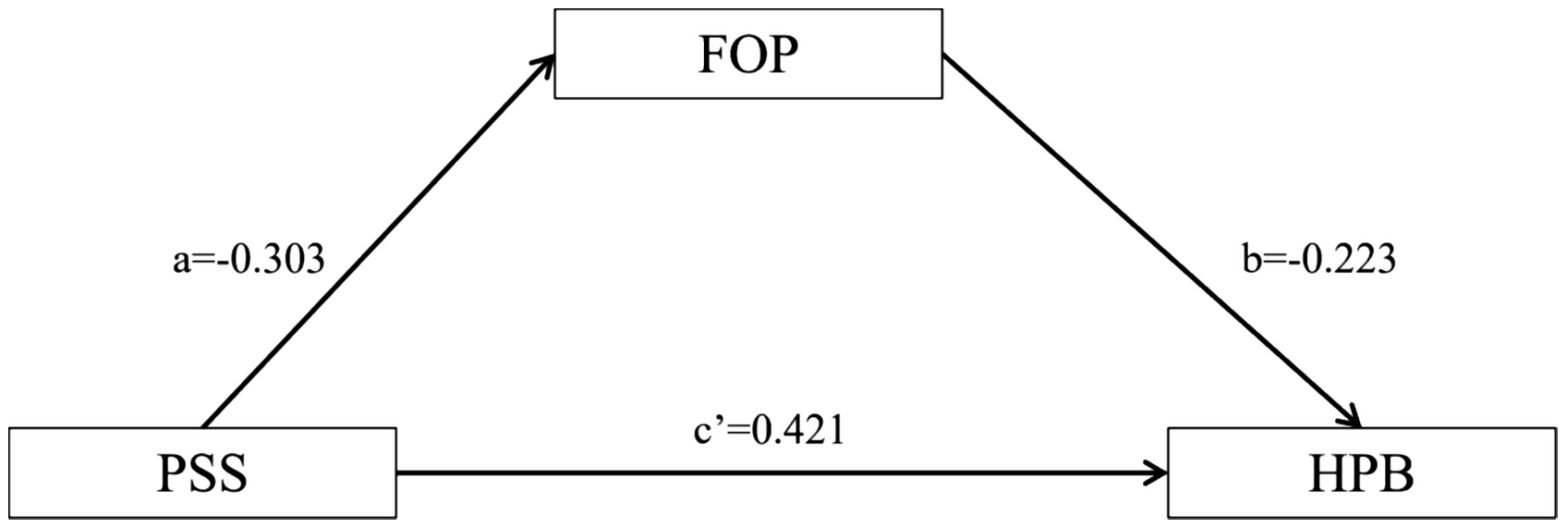
Schematic diagram of the mediating effect of FOP between PSS and HPB. All estimates were calculated after controlling for age, educational level, and medical payment methods. Total effect (c) = 0.488; Indirect effect (ab) = 0.068; Mediation proportion = 13.9%.

## 4 Discussion

This study describes the levels of HPB among middle-aged and young patients following PCI and employs a mediation model to analyze the mediating role of FoP between PSS and HPB. The results indicate that there are significant correlations among the three variables, and that FoP partially mediates the relationship between PSS and HPB (the mediation effect accounts for 13.9% of the total effect). This mediation model suggests that enhancing PSS may be associated with reduced FoP, which in turn improves HPB, providing a preliminary theoretical basis for the design of future longitudinal or intervention studies.

### 4.1 Current status of HPB among young and middle-aged patients following PCI surgery

The total score for HPB among young and middle-aged patients after PCI was 88.09 ± 9.98, with a mean item score of 2.20, which was below the midpoint of the 4-point scale (2.50). This suggests that the overall level of HPB in this group was below, but relatively close to, the midpoint of the scale, indicating room for further improvement. These findings are generally consistent with those of previous studies (10). Regarding associated factors, insufficient disease awareness and self-management skills may be linked to lower HPB. Some patients hold the misconception that “surgery equals a cure,” leading them to give low priority to long-term risk factor control and secondary prevention; this cognitive pattern may coincide with inadequate health behavior implementation (33). In addition, middle-aged and young patients are at a life stage where professional and family responsibilities converge; work-related stress, financial burdens, and social role conflicts are particularly pronounced, making it difficult to modify established lifestyle habits such as smoking, alcohol consumption, high-fat diets, and sedentary behavior (34). Some patients also restrict physical activity out of concern that exercise may trigger disease recurrence, and this anxiety is associated with lower rehabilitation behavior (35).

It is worth noting that, compared with older adult patients following PCI, middle-aged and young patients have better physical function but face greater occupational and family pressures, and their health behaviors are more easily influenced by a fast-paced work environment, time constraints, and social obligations (35). Older adult patients often have more time after retirement and more stable family care resources, so they tend to show higher adherence to medical advice, whereas middle-aged and young patients more frequently have difficulty maintaining healthy behaviors. Therefore, the lack of HPB among middle-aged and young patients is not only related to disease factors but also closely linked to the psychosocial characteristics of their life stage. This finding suggests that clinical practice should address the unique characteristics of this patient population by focusing on individualized health management and long-term behavioral support to explore feasible strategies for improving their adherence to health behaviors.

This study found that age, educational level, and medical payment methods are factors associated with HPB among young and middle-aged patients following PCI. Regarding age, middle-aged patients (45–59 years old) scored higher on health-promoting behaviors than younger patients (18–44 years old). Middle-aged patients demonstrated a stronger perception of disease risk and a greater sense of responsibility toward their family and health, which may be associated with higher levels of HPB. Previous studies have also shown that young patients often neglect health management due to a perception of being “young and asymptomatic (10).” Additionally, they face greater work-related stress, exhibit lower adherence to health behaviors, and may have their attention diverted from rehabilitation management by the need to return to work. Regarding educational level, patients with higher levels of education exhibited better health-promoting behaviors, consistent with previous research (36). Those with higher education possess greater health literacy and stronger abilities to access and interpret information; they are more inclined to proactively learn about rehabilitation and practice healthy behaviors. In contrast, those with lower educational level have weaker information-gathering capabilities and insufficient knowledge of disease and rehabilitation. Their health behaviors are more easily influenced by traditional beliefs, and they have a lower sense of personal control, making it relatively difficult to implement rehabilitation management. Regarding medical payment methods, patients covered by urban employee medical insurance exhibited better health-promoting behaviors than those covered by urban and rural resident medical insurance or self-paying patients. This may be related to the higher reimbursement rates under urban employee medical insurance, which may make it easier for them to afford postoperative follow-up visits and rehabilitation guidance. These findings suggest that clinicians should explore personalized health education and intervention plans tailored to differences in patient age, educational level, and health insurance type to enhance patients’ cognitive understanding and self-efficacy, thereby improving the health support system.

### 4.2 Correlation analysis of PSS, FoP, and HPB

This study found a significant positive correlation between PSS and HPB (*r* = 0.715, *p* < 0.01); that is, the higher the level of PSS among young and middle-aged patients following PCI, the higher their level of HPB, consistent with previous studies (37). In terms of underlying mechanisms, higher PSS is associated with lower negative emotions, such as postoperative anxiety and depression, which may support stronger recovery beliefs and more proactive behaviors, such as medication adherence, dietary control, and regular exercise (13). Furthermore, high perceived social support is often accompanied by a stronger sense of self-efficacy; the care and encouragement from family members, healthcare providers, and peers may increase control over rehabilitation behaviors and provide practical rehabilitation guidance and behavioral supervision (16). Previous studies have also confirmed that patients with high PSS are better able to integrate family, medical, and social resources and are more inclined to proactively adopt healthy lifestyles; conversely, patients with low PSS are more likely to experience reduced rehabilitation adherence, which is closely related to difficulties in maintaining HPB (38).

The results of this study indicate that PSS is significantly negatively correlated with FoP (*r* = −0.478, *p* < 0.01); that is, the higher the level of PSS, the lower the level of FoP, consistent with previous studies (18). Existing research suggests that patients undergoing cardiac surgery often experience FoP due to insufficient understanding of their prognosis, concerns that postoperative recurrence will affect their work and family responsibilities, and a lack of confidence in their recovery (39). In this study, higher levels of PSS were associated with lower levels of FoP, suggesting that the companionship and care of family members, professional guidance from healthcare providers, and the sharing of experiences among peers may help patients accurately understand their prognosis and alleviate feelings of uncertainty, thereby contributing to a reduction in FoP (40).

The results of this study indicate that FoP is negatively correlated with HPB (*r* = −0.610, *p* < 0.01). This suggests that, among middle-aged and young patients following PCI, lower FoP is associated with better HPB, consistent with previous research (41). FoP is a common negative psychological response among patients with chronic illnesses. While moderate FoP may encourage patients to take their disease management seriously, excessive FoP is often accompanied by negative emotions such as anxiety and helplessness, as well as low self-efficacy, and is associated with behaviors such as avoiding medication, refusing follow-up visits, and resisting regular exercise (21). Research indicates that excessive FoP often leads to negative emotions such as anxiety and helplessness, diminishes self-efficacy, and results in behaviors like medication avoidance, refusal of follow-up examinations, and resistance to regular exercise (42). These factors subsequently undermine the implementation and adherence to HPB. Furthermore, research indicates that excessive fear of disease progression disrupts patients’ psychological equilibrium, causing them to overemphasize negative disease-related information while overlooking the positive impact of healthy behaviors on disease control (43). This further diminishes their proactive engagement in recovery.

### 4.3 The mediating role of FoP in the relationship between PSS and HPB

The results of this study indicate a statistically significant indirect association of FoP with the relationship between PSS and HPB. Specifically, PSS was negatively associated with FoP, while FoP was negatively associated with HPB. Further mediation analysis showed that the indirect effect of FoP accounted for 13.9% of the total effect of PSS on HPB. These findings suggest that, in the study sample, higher levels of PSS were associated with lower levels of FoP and higher levels of HPB, and that FoP may partially account for the statistical association between PSS and HPB. PSS, as a positive social resource, may be related to the emotional support, informational support, and rehabilitation guidance available to patients, and may also be associated with their perceptions of disease-related information and psychological states (21). At the same time, higher levels of FoP were associated with lower levels of HPB (18). Consistent with social cognitive theory (22), individual behavior may be influenced by both social environmental and psychological factors. Higher levels of perceived social support may be associated with greater confidence in recovery and lower psychological stress, as well as lower levels of FoP (44). Conversely, lower levels of FoP may be associated with greater proactive engagement in rehabilitation and HPB (42). However, because this study employed a cross-sectional design, the temporal sequence among the variables could not be established. Therefore, these findings should be interpreted as statistical associations and an indirect effect pattern rather than as evidence of definitive causal relationships or underlying psychological mechanisms. Future longitudinal and intervention studies are warranted to further examine the temporal relationships and potential pathways among PSS, FoP, and HPB.

It is worth noting that the mediating effect of FoP accounted for 13.9% of the total effect in this study, suggesting that it partially mediated the relationship between PSS and HPB. Although this proportion is not high, given that HPB are influenced by a combination of physiological, psychological, and social factors, the fact that a single psychological factor can explain approximately 14% of the effect pathway remains clinically significant. This indicates that FoP is one of the key psychological mechanisms linking PSS to HPB, though it is not the sole mechanism. In other words, the positive effect of PSS on HPB among young and middle-aged patients after PCI is primarily achieved through direct pathways, but it may also exert influence through other mechanisms such as enhancing self-efficacy, improving disease cognition, and increasing treatment adherence. The findings of this study suggest that, in clinical practice, in addition to continuously strengthening the support systems of patients’ families, healthcare providers, and the broader social network, attention should also be paid to negative psychological states such as fear of disease progression. By implementing targeted psychological support and health education interventions, the formation and maintenance of healthy behaviors among patients can be further promoted, thereby enhancing long-term postoperative rehabilitation outcomes.

Based on the findings of this study, HPB among young and middle-aged patients after PCI were associated with demographic characteristics, PSS, and FoP. Accordingly, the following considerations may be relevant to clinical health management and may provide a reference for future personalized health management and intervention research. Given the cross-sectional design of this study, these suggestions should be considered as potential directions rather than evidence-based recommendations of effectiveness.

(1) Focus on populations with relevant characteristics and explore personalized health management strategies.

This study found that demographic characteristics, such as age and educational level, were associated with HPB. In clinical practice, healthcare providers may consider identifying patients with different health management needs based on characteristics such as age and educational level, and appropriately tailoring health education content, information presentation methods, and follow-up approaches. For example, online health education may be explored to accommodate the information-seeking patterns of younger patients, whereas patients with lower educational levels may benefit from more accessible and individualized health guidance.

(2) Emphasize social support and explore multidimensional support models.

This study found a positive association between PSS and HPB, suggesting that social support may be an important correlate of HPB. In clinical health management, attention may be given to patients’ family support, support from healthcare providers, and peer support. Strategies such as health education, family involvement, and ongoing follow-up may be explored to provide additional support for patients’ health management. Future intervention studies are needed to determine whether different support models can improve HPB.

(3) Address FoP and explore the integration of psychological support with health behavior management.

This study found that FoP was negatively associated with HPB and showed a statistically significant indirect association between PSS and HPB. Accordingly, clinical health management may consider assessing psychological experiences such as fear of disease progression and providing appropriate psychological support and health education. For patients with substantial psychological distress, referral to qualified mental health professionals for further assessment and intervention may also be considered. The effectiveness of such approaches should be further evaluated in future intervention studies.

(4) Strengthen continuous health management and further evaluate long-term management strategies.

Young and middle-aged patients after PCI may benefit from continued attention to long-term disease management and the maintenance of health-promoting behaviors. In addition to routine follow-up, healthcare providers may explore models of continuous health management, such as online follow-up, health education, and community-based support, according to patients’ needs and healthcare resources. Future longitudinal and intervention studies could further examine whether different management models are associated with sustained health-promoting behaviors and relevant clinical outcomes.

## 5 Strengths and limitations

This study possesses certain theoretical and practical advantages. First, grounded in social cognitive theory, it confirms the mediating role of FoP in the relationship between PSS and HPB among middle-aged and young patients after PCI. By clarifying the causal pathways among these three factors, it provides robust theoretical support for clinical health management of this patient group and expands the application of social cognitive theory in cardiovascular rehabilitation. Second, by focusing on the young-to-middle-aged PCI patient population, this study addresses the limitation of previous research, which predominantly targeted older adult patients. It specifically analyzes the health-related psychological and behavioral characteristics of this group, providing practical evidence for precision rehabilitation interventions and improving postoperative recovery quality. Additionally, this study simultaneously analyzed influencing factors such as age, educational level, and medical payment methods. By integrating these factors with the mediating mechanisms, it proposes comprehensive intervention recommendations, further enhancing the practical guidance and operational feasibility of the research findings.

This study has several limitations. First, convenience sampling was used, and only young and middle-aged patients who had undergone PCI at a single tertiary Grade A hospital in Sichuan Province were included. The relatively narrow sampling frame may have introduced selection bias. The participants may have been more likely to have relatively stable medical conditions, better cognitive functioning, and higher survey compliance; therefore, the representativeness and generalizability of the findings to young and middle-aged patients undergoing PCI in primary care settings, community settings, and other regions may be limited. Future studies could adopt multicenter, large-sample designs and use consecutive enrollment, stratified sampling, or probability sampling to improve sample representativeness and the external validity of the findings.

Second, this study employed a cross-sectional design, with all variables measured at a single time point. Therefore, the temporal sequence among PSS, FoP, and HPB could not be established. Although the mediation analysis identified a statistically significant indirect effect of FoP, this finding reflects a statistical association among the variables and does not support causal inference. Future longitudinal, cross-lagged, or intervention studies could further examine the temporal relationships and potential pathways among these variables.

Third, all variables were assessed using patient self-report questionnaires, which may be subject to common-method bias and social desirability bias. Although the Harman single-factor test did not indicate substantial common-method bias, this approach is only a preliminary assessment and cannot completely exclude such bias. Because PSS, FoP, and HPB were all self-reported by the same participants at the same time point, shared response tendencies may have influenced the observed associations among variables and the estimation of the indirect effect. In addition, HPB may be subject to social desirability, and participants may have overreported their actual behavioral levels. Therefore, the indirect effect of FoP identified in this study should be interpreted with caution. Future studies could incorporate multi-source and multi-time-point assessments, together with objective indicators such as step counts or physical activity monitoring, medication-use records, and follow-up data, to reduce potential reporting and measurement biases.

Finally, this study adjusted for only selected demographic and disease-related variables and did not include several potentially relevant factors, such as postoperative cardiac function classification, comorbidity severity, use of antidepressants or anxiolytics, self-efficacy, and health literacy. These factors may be associated with both FoP and HPB and may have contributed to residual confounding in the observed associations and the estimation of the indirect effect. Future studies should consider incorporating a broader range of clinical, treatment-related, and psychosocial variables to further improve the robustness and interpretability of the findings.

## 6 Conclusion

In summary, there is still room for further improvement in HPB among middle-aged and young PCI patients in China; there is a statistically significant association among perceived social support, fear of disease progression, and HPB, and FoP exerts a partial mediating effect in the relationship between PSS and HPB. Based on cross-sectional data analysis, this study reveals the associative characteristics and potential mechanisms among these three factors, providing a reference for clinical healthcare professionals to implement individualized interventions. Future efforts may focus on enhancing patients’ PSS and alleviating FoP, thereby improving HPB and rehabilitation outcomes among middle-aged and young PCI patients.

## Data Availability

The original contributions presented in the study are included in the article/Supplementary material, further inquiries can be directed to the corresponding authors.
